# Local Translation Across Neural Development: A Focus on Radial Glial Cells, Axons, and Synaptogenesis

**DOI:** 10.3389/fnmol.2021.717170

**Published:** 2021-08-09

**Authors:** Manasi Agrawal, Kristy Welshhans

**Affiliations:** ^1^School of Biomedical Sciences, Kent State University, Kent, OH, United States; ^2^Department of Biological Sciences, University of South Carolina, Columbia, SC, United States

**Keywords:** local translation, neurodevelopment, neurodevelopmental disorders, axon growth and guidance, synaptogenesis, radial glial cells, growth cone

## Abstract

In the past two decades, significant progress has been made in our understanding of mRNA localization and translation at distal sites in axons and dendrites. The existing literature shows that local translation is regulated in a temporally and spatially restricted manner and is critical throughout embryonic and post-embryonic life. Here, recent key findings about mRNA localization and local translation across the various stages of neural development, including neurogenesis, axon development, and synaptogenesis, are reviewed. In the early stages of development, mRNAs are localized and locally translated in the endfeet of radial glial cells, but much is still unexplored about their functional significance. Recent *in vitro* and *in vivo* studies have provided new information about the specific mechanisms regulating local translation during axon development, including growth cone guidance and axon branching. Later in development, localization and translation of mRNAs help mediate the major structural and functional changes that occur in the axon during synaptogenesis. Clinically, changes in local translation across all stages of neural development have important implications for understanding the etiology of several neurological disorders. Herein, local translation and mechanisms regulating this process across developmental stages are compared and discussed in the context of function and dysfunction.

## Introduction

Neurons are highly polarized cells with extensive spatial compartmentalization. The long and complex nature of these cells requires that distal axons and dendrites respond to external cues rapidly, without direct communication with the soma. During neural development, neurons are born, migrate, extend processes, and form synapses to form a functional nervous system. Even during these early stages, many signaling cascades take place at locations distant from the soma. A key mechanism regulating this dynamic signaling is the localization of translational machinery, including mRNA transcripts, ribosomes, and RNA-binding proteins (RBPs), to subcellular regions of developing neurons (Jung et al., 2014; Holt et al., 2019). In response to specific cues or demands, mRNAs undergo coordinated local translation. RBPs are key players in this process, which function by recognizing and binding to one or more sequences on their target mRNAs, forming ribonucleoprotein (RNP) complexes (Glisovic et al., 2008; Czaplinski, 2014; Fernandopulle et al., 2021). This complex regulates multiple post-transcriptional functions, including splicing, mRNA localization, and translation.

For many years, there was controversy in the field about whether local translation occurred in axons. However, the literature is now clear that distal developing axons contain the machinery needed to translate mRNAs, including mRNAs, ribosomes, and translation initiation factors (Bassell et al., 1998; Campbell and Holt, 2001; Zhang et al., 2001; Zivraj et al., 2010). Furthermore, mRNAs are clearly translated in both developing and mature axons (reviewed in Dalla Costa et al., 2020; Kim and Jung, 2020). Due to the distal nature of axons and recent advances in sequencing techniques, our knowledge about axonal transcriptomes and translational machinery has greatly increased. These studies have demonstrated that there is remarkable diversity in both the abundance and function of these transcriptomes, depending on the neuronal type and developmental stage. Furthermore, these studies have begun to provide insight into how the axonal transcriptome is dysregulated in neurological disorders.

The development of a functional nervous system involves many tightly orchestrated steps, including differentiation, neurogenesis, axon growth and guidance, axon branching, and synaptogenesis. During early neurogenesis, radial glial cells, the proliferative neural stem cells that line the ventricular surface and extend to the pial lamina, undergo symmetric and asymmetric cell division, which leads to an increase in the number of radial glial cells and neurons (Noctor et al., 2001, 2008). Both intrinsic and extrinsic cues regulate the migration of newly born neurons along their long radial fibers. After neurons undergo polarization, axons extend and are tipped with sensory and motor pathfinding structures termed growth cones. Growth cones contain receptors for guidance cues that can be attractive or repulsive. Guidance cue ligand binding to receptors leads to activation of intracellular signaling pathways, including local translation (Stoeckli, 2018). The ultimate outcome of this signaling is remodeling of the growth cone cytoskeleton and/or changes in adhesion, which results in steering toward the appropriate synaptic target (Gallo and Letourneau, 2004; Kalil and Dent, 2005; Vitriol and Zheng, 2012; Omotade et al., 2017). After reaching their postsynaptic partners, growth cones form pre-synaptic terminals, characterized by the presence of synaptic vesicles, and undergo synaptogenesis.

Changes in proteomes, in response to intrinsic cellular functioning and environmental stimuli, are necessary to tightly regulate these processes. In this review, we present and assess current knowledge about localized transcriptomes and associated translational regulation during each of these developmental stages. During the stages of guidance and synaptogenesis, we focus on axons and the formation of presynaptic terminals; local translation in dendrites is beyond the scope of this review. We also discuss key findings evaluating the importance of local translation in the development of a functional nervous system. Additionally, we highlight how dysregulation of these processes can lead to neurological disorders.

## Local Translation in Radial Glial Cells During Neurogenesis

**FIGURE 1:**
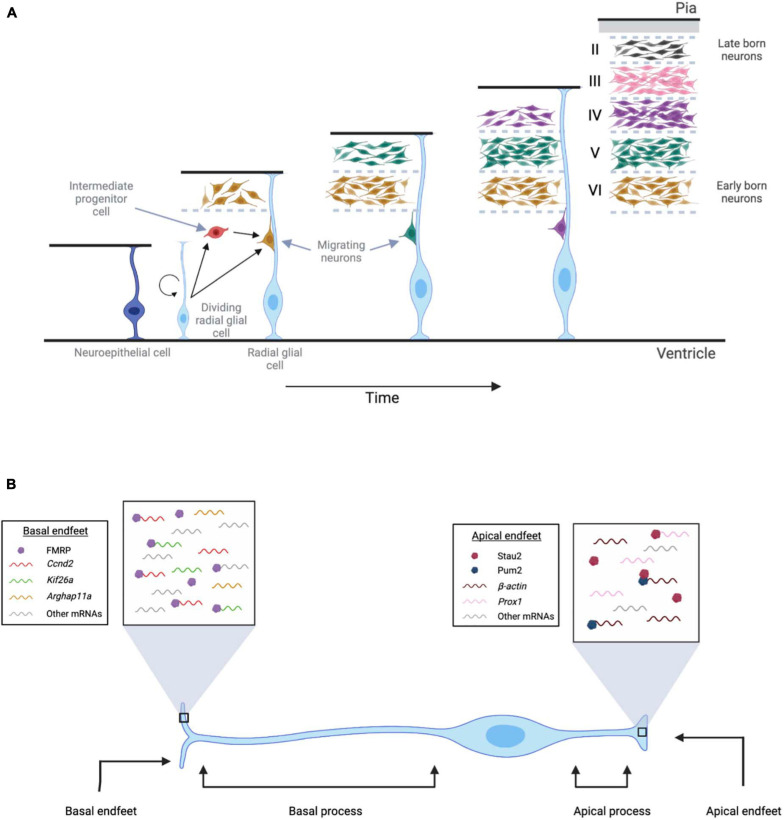
Localization of mRNAs and RBPs in radial glial cells during neurogenesis. **(A)** Neuroepithelial cells (dark blue) undergo symmetric division before neurogenesis begins and give rise to a pool of neural progenitor cells. During neurogenesis, these neuroepithelial cells transition into radial glial cells (light blue), which have a longer process extending toward the pial region and a shorter process extending toward the ventricular surface. As development progresses, radial glial cells can divide either symmetrically or asymmetrically to form more radial glial cells, intermediate progenitor cells (red), and neurons. Using radial glial cells as a scaffold, newly born neurons migrate toward the pial surface, forming cortical layers II-VI. **(B)** FMRP, Stau2, and Pum2 localize mRNAs to radial glial cell endfeet. In the basal endfeet, local translation of *Ccnd2* and *Kif26a* mRNA likely regulates cell fate and microtubule dynamics. *Arhgap11a* mRNA also localizes in the basal endfeet, and its local translation contributes to the laminar organization of the developing cortex. In the apical endfeet, the local translation of β*-actin* and *prox1* mRNAs contribute to cell fate determination and cytoskeletal remodeling.

Neuroepithelial cells undergo symmetric division before neurogenesis begins, giving rise to an increase in the number of neural progenitor cells (Rakic, 1995). During neurogenesis, these neuroepithelial cells transition into radial glial cells, neural stem cells that generate neurons in the developing cerebral cortex (Figure 1A; Malatesta et al., 2000; Noctor et al., 2001). Radial glial cells are characterized by their bipolar morphology: the long process, extending toward the pial surface, forms the basal region and consists of the basal process and the basal endfeet. The shorter process, extending toward the ventricular surface, forms the apical region and consists of the cell body, nucleus, and apical endfeet (Misson et al., 1988; Noctor et al., 2001, 2002; Götz and Huttner, 2005).

As development progresses, radial glial cells can divide either symmetrically or asymmetrically to form more radial glial cells, intermediate progenitor cells, and neurons (Götz and Huttner, 2005; Huttner and Kosodo, 2005; Tsunekawa et al., 2014). The division plane for asymmetric cell division can result in an uneven distribution of the apical and basal processes, such that each daughter cell receives one of these processes (Miyata et al., 2001; Noctor et al., 2001; Kosodo et al., 2004; Tsunekawa et al., 2014). This resulting unequal dissemination of molecular machinery to the daughter cells can direct cell fate (Knoblich, 2008, 2010; Tsunekawa et al., 2014).

The basal endfeet interact with the basal lamina and extrinsic cues present in the pial surfaces using filopodia-like protrusions. Recent studies have established that mRNAs are actively localized to basal endfeet, resulting in a specific transcriptome that is locally translated (Pilaz et al., 2016). Using *in situ* hybridization and RNA immunoprecipitation followed by microarray (RIP-Chip), many mRNA transcripts have been identified in the radial glial cell endfeet, including those that encode proteins crucial for neurogenesis and basal endfeet remodeling (Tsunekawa et al., 2012, 2014; Pilaz et al., 2016, 2020). Although much is still unknown about this novel area of research, local translation could be stimulated either intrinsically and/or via signaling from the pial surface through filopodial protrusions, thereby regulating neurogenesis and cortical expansion.

mRNAs are actively and rapidly transported to the basal endfeet from the cell body with the help of *cis*- and *trans*-acting elements, including RBPs (Pilaz et al., 2016; D’arcy and Silver, 2020). Although this is a relatively new area of study, a few RBPs that regulate local protein synthesis have been identified in radial glial cells, including Fragile X mental retardation protein (FMRP), Staufen 2 (Stau2), and Pumilio2 (Pum2) (Vessey et al., 2012; Pilaz et al., 2016). A recent study demonstrated that FMRP actively moves in radial glial cells and is enriched in the basal endfeet (Pilaz et al., 2016). Using RIP-Chip, the presence of more than 115 mRNA transcripts, including members of the kinesin family, zinc finger transcripts, and signaling molecules, that bind specifically to FMRP in the basal endfeet, were identified (Pilaz et al., 2016). However, any other mRNAs that localize in basal endfeet, independent of FMRP, have yet to be identified.

FMRP binds to Cyclin D2 (*Ccnd2*) mRNA and kinesin family member 26a (*Kif26a*) mRNA (Pilaz et al., 2016), and the 3′UTRs of *Ccnd2* and *Kif26a* mRNAs are necessary for their localization to the basal endfeet (Tsunekawa et al., 2012; Pilaz et al., 2016; Figure 1B). Cyclin D2 is a cell cycle regulator that controls the proliferation of intermediate progenitor cells (Glickstein et al., 2009). Using fluorescent reporters, *Ccnd2* mRNA has shown to be actively transported in radial glial cells and locally translated in endfeet (Pilaz et al., 2016). Functionally, the localization of Cyclin D2 protein during asymmetric division of the neural progenitor cell is critical. Cyclin D2 protein is inherited only by the daughter cell that becomes a progenitor cell, thereby maintaining its proliferative state (Tsunekawa et al., 2012, 2014). If Cyclin D2 protein is depleted in the daughter cell, then this results in the formation of a post-mitotic neuron. Additionally, overexpression of Cyclin D2 leads to an increase in the number of neural progenitor cells, thus confirming its role in neurogenesis (Saffary and Xie, 2011; Tsunekawa et al., 2012). KIF26a, an unconventional kinesin of the kinesin 11 family, binds to microtubules and plays a role in nervous system development (Zhou et al., 2009). The 3′UTR of *Kif26a* mRNA is sufficient for its localization and it is locally translated in the basal endfeet (Pilaz et al., 2016). More research is needed in this area, but the local translation of KIF26A likely regulates the microtubule movement that is needed for radial glial cell developmental processes. Future studies should provide greater insight into the functional requirements of these locally translated mRNAs for neurogenesis.

Another protein that is enriched in the basal endfeet and regulates radial glial cell morphology is a RhoA GAP (GTPase-activating protein), called Arhgap11A. It is known to modulate cytoskeletal dynamics by promoting GTP hydrolysis and can regulate complex branching in the basal endfeet of radial glial cells (Pilaz et al., 2020). The 5′UTR of *Arhgap11a* mRNA mediates both its active transport to the basal process and its local translation in the basal endfeet (Pilaz et al., 2020). Interestingly, this study also showed that loss of Arhgap11A affects not only basal process morphology and endfeet branching, but also radial migration of neurons and overall laminar organization of the developing cortex. Thus, multiple lines of evidence are emerging to support the novel concept of local translation in radial glial cells.

Although most studies in this area have focused on the RNA localization and local translation in the basal process of radial glial cells, there has been some development in identifying transcripts that undergo local translation at the apical end, near the ventricular surface (Figure 1B). Stau2, an RBP involved in mRNA localization, is enriched in the apical region of radial glial cells (Vessey et al., 2012). Here, it colocalizes with a tight junction protein, called ZO-1, which is also an apical marker (Vessey et al., 2012). Stau2 binds to β*-actin* and *prox1* (Prospero homeobox protein 1) mRNAs. Prox1 regulates cell fate determination, and β-actin is necessary for cytoskeletal remodeling. Using an shRNA knockdown approach, Vessey et al. (2012) found that Stau2 is important for the maintenance of the neuronal progenitor population (Vessey et al., 2012). Interfering with the interaction of Stau2 with *prox1* mRNA results in delocalization of *prox1* mRNA, an increase in Prox1 protein, and premature differentiation of progenitor cells into neurons. Collectively, these results suggest that the regulation of *prox1* mRNA translation by Stau2 is important for appropriate neurogenesis and differentiation.

Taken together, these studies discover that both the basal and apical regions of radial glial cells are enriched with mRNA transcripts that are locally translated. Data are rapidly emerging that mRNA transcripts are actively transported along the basal process, and it will be interesting to determine how mRNAs are localized in apical processes as well (Tsunekawa et al., 2012, 2014; Pilaz et al., 2016, 2020; Pilaz and Silver, 2017). mRNA localization and local translation in the endfeet appear to be critical for regulating the production of progenitor cells and neurons and regulating cytoskeletal dynamics, thereby playing a significant role in cortical development. However, these studies focus on a limited number of elements. There is currently a large gap in our knowledge about the mRNA localization motifs that are involved and the modes of mRNA localization in radial glial cells. Although untranslated regions are considerably longer in transcripts localized in neurons and neuronal processes (Miura et al., 2013; Taliaferro et al., 2016; Tushev et al., 2018), it is unknown how extensively this applies to localized transcripts in radial glial cells. Furthermore, only a limited number of RBPs have been identified, and very little is known about other factors that may regulate translation in radial glial cell endfeet, such as ribosomes, eukaryotic initiation factors and non-coding mRNAs. Future studies will need to provide a more complete picture of the mRNAs in both apical and basal processes and their interactions with RBPs. This is a very young, but promising area of investigation, and thus, much more research is needed to also understand the functional relevance of local translation for neurogenesis and neuronal differentiation in the developing cortex.

## Local Translation During Axon Growth and Guidance

Growth cones, present at the tip of developing axons, are highly motile structures that sense and respond to extracellular guidance cues. This guidance cue-mediated pathfinding directs growth cones to their synaptic targets. Recent advancements in cellular and molecular techniques, such as isolation of axonal and growth cone fractions and next-generation sequencing, have identified many mRNAs that localize in developing axons and may be locally translated in response to guidance cues. RBPs bind to mRNAs to form RNP complexes, which help mediate the axonal transport of these mRNAs. This transport is facilitated either by direct binding of the RNPs to motor proteins or by hitchhiking on organelles, such as endosomes or lysosomes (Cioni et al., 2018, 2019; Sahoo et al., 2018; Liao et al., 2019). mRNAs localized to developing axons encode many functional classes of proteins, including cell adhesion molecules, guidance receptors, cytoskeletal components, and members of signaling cascades (Donnelly et al., 2013; Batista and Hengst, 2016; Cioni et al., 2018). Our knowledge about the role of local translation in axon growth and guidance is much more extensive as compared to that during neurogenesis. This field has identified numerous mRNAs that are locally translated during axon development. However, this field has also gone beyond identification in recent years to elucidate many novel mechanisms that contribute to the process of mRNA localization and translation. Here, we review this literature showing that local translation of mRNAs in axons and growth cones contributes to the formation of precise neural connections during development.

### Mechanisms Underlying Localization and Local Translation of β-Actin mRNA

The local translation of β-actin, a cytoskeleton protein that is critical to the process of growth cone pathfinding, has been a major area of focus in the axon guidance field. The first evidence that β*-actin* mRNA might be locally translated within growing axons used *in situ* hybridization to show that it is enriched within the periphery of growth cones (Bassell et al., 1998). The distribution of β*-actin* mRNA within growth cones occurred in a morphology-dependent manner, such that more β*-actin* mRNA was seen in growth cones that had a flattened lamellar structure with defined central domain and peripheral regions, as compared to spindle-shaped growth cones. β*-actin* mRNA also colocalized with elements of the polyribosome complex and microtubules within growth cones, suggesting that it could be translated locally.

**FIGURE 2:**
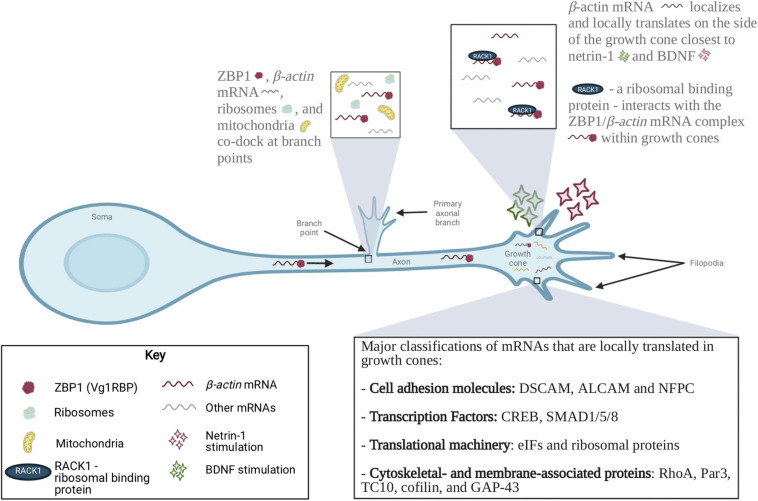
Localization of mRNAs and RBPs in axons and growth cones during axon growth, guidance and branching. mRNAs localized to developing axons encode many functional classes of proteins, including cell adhesion molecules, guidance receptors, cytoskeletal components, and members of signaling cascades. The local translation complex of β*-actin* mRNA, ZBP1, ribosomes and mitochondria is involved in axon branching. BDNF and netrin-1 induced local translation of β*-actin* mRNA is also involved in axon guidance. Four major classes of mRNAs that are locally translated in growth cones are cell adhesion molecules, transcription factors, translational machinery and cytoskeletal- and membrane-associated proteins. The local translation of these proteins is a tightly regulated process that is critical for the formation of appropriate neural networks.

Numerous studies have since built on this foundational work to demonstrate that β*-actin* mRNA is locally translated in growth cones and regulates axon guidance. Neurotrophin-3, which stimulates axon growth, results in increased levels of β*-actin* mRNA within axons of developing neurons (Zhang et al., 1999, 2001). Additionally, treatment with antisense oligonucleotides to the β-actin zipcode, a sequence in the 3′UTR of β*-actin* mRNA necessary for its localization, leads to retractive growth cone behavior, as opposed to the forward movement observed with control oligonucleotides (Zhang et al., 2001). Two key studies then showed that asymmetric local synthesis of β*-actin* mRNA on the side of the growth cone closer to the attractive guidance cue (either BDNF or netrin-1) is necessary for axon guidance (Leung et al., 2006; Yao et al., 2006; Figure 2). Using retinal ganglion cells from *Xenopus* and a photoconvertible translation reporter, Leung et al. (2006) showed that netrin-1 results in an increase in β*-actin* translation in growth cones (Leung et al., 2006). Inhibition of β*-actin* mRNA translation prevented netrin-1 induced attractive growth cone turning, confirming that local translation controls axon guidance. Using BDNF, similar results were shown by Yao et al. (2006): β*-actin* mRNA, zipcode binding protein 1 (ZBP1), and β-actin protein are asymmetrically distributed on the side of the growth cone closest to the attractive guidance cue BDNF (Yao et al., 2006). In addition, treatment with antisense oligonucleotides to prevent β*-actin* mRNA from binding to ZBP1 prevented BDNF-induced attractive growth cone turning. Together, these two studies provided the first evidence that local translation is necessary for appropriate axon pathfinding in the developing nervous system.

Down syndrome critical region 1 (DSCR1) also mediates BDNF-induced axonal pathfinding during development by regulating local translation of mRNAs and actin dynamics (Wang et al., 2012, 2016). DSCR1 localizes in the axons of hippocampal neurons and helps mediate the local translation of β*-actin* mRNA (Wang et al., 2016). Overexpression of DSCR1, which occurs in Down syndrome, results in increased local translation of β*-actin* mRNA. Similarly, neurons deficient in DSCR1 showed reduced β*-actin* mRNA translation and impaired growth cone turning. Mechanistically, it is thought that DSCR1 controls the phosphorylation of FMRP, which then regulates BDNF-dependent translation of β*-actin* mRNA. Thus, these results indicate that DSCR1 plays an important role in regulating the local protein synthesis of β*-actin* mRNA, which is central to axonal pathfinding.

More recent studies have provided additional details about the dynamics of β*-actin* mRNA in developing axons. Under basal conditions, the majority of β*-actin* mRNA molecules are located as single copies in developing axons, and most growth cones contain fewer than four copies of this mRNA (Turner-Bridger et al., 2018). Future studies will need to provide additional information about how growth and guidance cues affect this process. That is, does stimulation result in multiple copies of β*-actin* mRNA being localized and translated together? Furthermore, does stimulation affect the copy number of mRNAs within growth cones? An additional study has started to provide some insight in relation to mRNA transport: β*-actin* mRNA undergoes bidirectional trafficking within retinal ganglion cell axons in a microtubule-dependent manner. Stimulation with netrin-1 elicits increased β*-actin* mRNA movement in the anterograde direction in axons, and from the central domain to the periphery within growth cones (Leung et al., 2018). Interestingly, this process is quite rapid. The polarization of β*-actin* mRNA to the near-side of the growth cone can be visualized within 1–2 min of exposure to netrin-1 (Leung et al., 2018). Using single-molecule translation imaging in *Xenopus* retinal ganglion cells, Ströhl et al. (2017) demonstrated that there is a burst of β-actin translation within 20 s after netrin-1 stimulation. Together, these findings elucidate that β*-actin* mRNA is localized and translated extremely quickly in response to a stimulus.

Zipcode-binding protein 1 (ZBP1) was identified as a 68 kDa mRNA-binding protein that binds to a 54 nucleotide zipcode sequence present in the 3′UTR of β*-actin* mRNA (Ross et al., 1997). In neurons, ZBP1 transports β*-actin* mRNA from the soma to the axonal shaft and growth cone (Zhang et al., 2001; Leung et al., 2006; Yao et al., 2006; Welshhans and Bassell, 2011). A study using antisense oligonucleotides to the 3′UTR zipcode sequence has confirmed the role of the 3′UTR in chemoattractant-mediated growth cone steering in *Xenopus* axons (Yao et al., 2006). ZBP1 itself is also necessary for axon guidance (Welshhans and Bassell, 2011). In cortical neurons isolated from ZBP1 knockout mice, growth cone turning, in response to either BDNF or Netrin-1, is impaired. Further, ZBP1 knockout mice show a loss of both BDNF and netrin-1 induced localization of β*-actin* mRNA, and stimulated local translation in cortical neuron growth cones (Welshhans and Bassell, 2011). A study by Kalous et al. (2014) found similar results, wherein netrin-1 stimulated attractive growth cone behavior *in vitro* is altered when a dominant-negative form of ZBP1, which lacks the RNA-binding domain, is expressed in *Xenopus* retinal ganglion cells (Kalous et al., 2014). Taken together, ZBP1 controls the local translation of β*-actin* mRNA to regulate axon guidance. However, it is important to note that Kalous et al. (2014) also showed that long-range navigation from the retina to the optic tectum *in vivo* was normal in dominant negative ZBP1 cells, despite the deficits observed *in vitro*. Thus, more research is needed to better understand the role of local translation *in vitro* versus *in vivo*, and whether this finding holds when examining other developing brain areas.

Another key player in the regulation of β*-actin* mRNA translation within growth cones is receptor for activated C kinase 1 (RACK1). RACK1 is a ribosomal binding protein that interacts with the mRNA/ZBP1 complex and regulates the local translation of β*-actin* mRNA (Ceci et al., 2012). Within growth cones of developing mouse cortical neurons, RACK1 and β*-actin* mRNA colocalize with point contacts, adhesion sites that link the actin framework in growth cones with the extracellular matrix (Kershner and Welshhans, 2017). Point contacts are central to axon growth and guidance in the developing nervous system (Gomez et al., 1996; Myers and Gomez, 2011; Myers et al., 2011). In line with this role, RACK1 knockdown in embryonic mouse cortical neurons impairs axon growth and guidance (Kershner and Welshhans, 2017; Kershner et al., 2019). Furthermore, colocalization of RACK1 and β*-actin* mRNA with point contacts increases following BDNF stimulation (Kershner and Welshhans, 2017), which results in the local translation of β*-actin* mRNA (Welshhans and Bassell, 2011). Thus, these data suggest that local translation of β*-actin* mRNA may occur at adhesion sites within growth cones (Kershner et al., 2019). Taken together, adhesion sites may be a direct link between extracellular stimuli and intracellular translational machinery, and thus play a crucial role in the local synthesis of β*-actin* mRNA to regulate axonal pathfinding during development.

### Major Classifications of mRNAs That Are Locally Translated in Growth Cones

In this section, we provide an overview of several studies that have provided large data sets about the local transcriptome and proteome in developing axons. High-throughput sequencing has allowed multiple studies to identify the local transcriptome in axonal growth cones. A total of 958 transcripts, of which 444 have known functions, were identified in *Xenopus* retinal ganglion cell growth cones using laser capture microscopy combined with microarray analysis (Zivraj et al., 2010). Many functional categories were identified in this microarray, including protein synthesis (e.g., RBPs, translation initiation factors, and elongation factors), transcription factors, proteins involved in cytoskeletal and motor functions, and intracellular signaling. This study also found that the number and complexity of mRNAs enriched within growth cones increases with developmental age. Earlier-stage growth cones that are involved in axon pathfinding are more enriched in transcripts related to protein synthesis and cytoskeletal dynamics. This was compared to later-stage growth cones, which were isolated 24 h later (i.e., around the time they would be reaching their synaptic targets). These later-stage growth cones are more enriched in transcripts related to cellular signaling and synaptic transmembrane proteins.

A similar study carried out in embryonic and adult dorsal root ganglion (DRG) axons using microarray analyses also identified differences in transcriptomes that were dependent on the developmental stage (Gumy et al., 2011). Axons from embryonic DRGs are enriched in mRNAs that encode cytoskeletal-related proteins and microtubule-associated proteins, which are necessary for axon growth and guidance; this is contrasted with adult DRGs, which are enriched in mRNAs encoding immune molecules related to nociception. However, some transcripts, including those related to protein synthesis and mitochondrial function, were enriched in both embryonic and adult DRGs. The changing axonal transcriptome over developmental time has also been shown using axon-TRAP (translating ribosome affinity purification), which identifies ribosome-bound mRNAs in axons. This technique was applied to characterize mRNAs present in distal compartments of mouse retinal ganglion cells *in vivo* at different stages of development, including pathfinding, branching, synaptogenesis, and following the creation of a mature synapse (Shigeoka et al., 2016). Similar to earlier studies, axons in early development show enrichment for mRNAs involved in axon extension and pathfinding, whereas later stage axons are enriched for mRNAs encoding proteins involved in dendrite formation, synapse formation, and synaptic transmission. Taken together, this suite of studies has clearly demonstrated, both *in vitro* and *in vivo*, that the axonal proteome changes throughout development and into adulthood to optimally support the changing needs of the axon and synapse. However, it is also of interest for future research to identify similarities between these multiple large-scale studies. That is, are some mRNAs found in axons at all developmental time points and in all cell types? This type of information is important to better understand the functionality of the local translatome.

A more recent study has focused on a single developmental stage to examine how specific guidance cues regulate the nascent local proteome. Using ultrasensitive proteomics that combines pulsed stable isotope labeling of amino acids in cell culture (pSILAC) and single-pot solid-phase-enhanced sample preparation (SP3), Cagnetta et al. (2018) found that there are distinct changes in the local proteome in the axons of *Xenopus* retinal ganglion cells in response to different guidance cues (Cagnetta et al., 2018). Under basal conditions, gene ontology analysis revealed that the most prominent nascent proteins enriched within developing axons encoded cell adhesion, extracellular matrix, cytoskeletal and ribosome-related proteins. Interestingly, different guidance cues (e.g., netrin-1, BDNF, Sema3A) stimulated the translation of a distinct set of nascent proteins. Overall, these large-scale studies have provided important information about the transcriptome and nascent proteome in developing axons and growth cones, and how this is affected by developmental time and extracellular guidance cues. In the next section of this review, we focus on additional studies examining four functional categories that these large scale studies have shown are enriched in developing axons and growth cones: cell adhesion molecules, transcription factors, protein synthesis machinery, and cytoskeletal-related proteins (Figure 2). We provide evidence from the literature that proteins in these categories are locally translated, and that this translation is functionally relevant for developing axons.

#### Cell Adhesion Molecules

Cell adhesion molecules (CAMs) are proteins shown to be involved in cell recognition, adhesion, axonal navigation, and cell migration. Three CAMs are locally translated and involved in axon growth and guidance: Down syndrome cell adhesion molecule (DSCAM), activated leukocyte cell adhesion molecule (ALCAM), and NF-protocadherin (NFPC). DSCAM is a receptor for netrin-1 and regulates axon growth and guidance (Garrett et al., 2012). *Dscam* mRNA colocalizes with two RBPs, FMRP and CPEB1 (cytoplasmic polyadenylation element binding protein 1), and is localized and locally translated in growth cones of developing mouse hippocampal neurons (Jain and Welshhans, 2016). Interestingly, netrin-1 stimulation for 20 min results in a decrease in *Dscam* mRNA, and an increase in DSCAM protein within growth cones. This local translation appears to be important for neuronal development because overexpression of DSCAM results in the stunting of axon length. ALCAM regulates axon elongation and fasciculation through homophilic and heterophilic interactions. It is locally translated within retinal ganglion cell growth cones *in vitro*, and this is dependent on ERK and TOR activation (Thelen et al., 2012). This local translation is functionally relevant because the axon length of neurons cultured on laminin and ALCAM is reduced by antisense oligonucleotides that target regions necessary for the local translation of ALCAM. These data suggest that locally translated ALCAM is necessary for axon growth. NFPC, a member of the cadherin superfamily, plays an important role in retinal ganglion cell axon guidance. Retinal ganglion axons must pathfind to their synaptic target, which is the tectum, and NFPC local translation in the mid-optic tract regulates this process (Leung et al., 2013). NFPC is locally synthesized in growth cones in response to Semaphorin 3A (Sema3A), a guidance cue that binds to neuropilin-1, specifically in the mid-optic tract. Disruption of neuropilin-1 signaling prevents NFPC local translation. Furthermore, disruption of NFPC alters appropriate axon guidance in the mid-optic tract *in vivo*. Taken together, these three studies have shown that controlled local translation of CAMs is essential to the formation of an appropriate neuronal network.

#### Transcription Factors

Transcription factors are also locally translated in developing axons. NGF stimulates the local translation of cAMP-response element binding protein (CREB) in axons (Cox et al., 2008). This locally translated CREB is then retrogradely transported to the nucleus to induce CRE-dependent transcription. Functionally, loss of axonally translated CREB leads to a decrease in neuronal survival, which suggests that anti-apoptotic genes are the transcriptional target of this pathway. Differentiation of neurons into specific subtypes is also regulated by local translation of transcription factors in axons. SMAD1/5/8 is a transcription factor that is locally translated in trigeminal ganglia axons in response to BDNF (Ji and Jaffrey, 2012). SMAD1/5/8 is then retrogradely trafficked to the nucleus by BMP4-induced signaling endosomes. SMAD1/5/8-initiated transcription regulates the differentiation of these trigeminal ganglion neurons into the ophthalmic and maxillary subtypes. Thus, these studies suggest that axonally translated transcription factors are important contributors to the developing nervous system.

#### Translational Machinery

Transport and local translation of mRNAs within the long processes of neurons requires a robust regulation of the translational machinery. Previous studies have shown that eukaryotic ribosomal assembly takes place mostly in the nucleolus. However, recent studies suggest that locally translated translational machinery and ribosomal proteins can be recruited to existing ribosomal subunits to maintain their physiological functions (Kar et al., 2013; Cagnetta et al., 2018; Shigeoka et al., 2019). In rat sympathetic neurons, the eukaryotic translation initiation factors, eIF2B2 and eIF4G2, are locally translated (Kar et al., 2013). Knockdown of eIF2B2 or eIF4G2 using siRNAs, only in the axonal compartment, decreases nascent protein synthesis within axons, including that of β*-actin* mRNA. Knockdown of eIF2B2 or eIF4G2 also inhibits axon growth. These findings suggest that local translation of these translation initiation factors is critical to support local translation and axon growth.

Assembly of locally translated ribosomal proteins on existing ribosomal subunits can occur in a nucleolus-independent manner. This was confirmed by using live-cell imaging and subcellular proteomics to show that ribosomal proteins can be locally translated in axons and integrated into pre-existing axonal ribosomes (Shigeoka et al., 2019). A specific *cis*-element upstream of the initiation codon (termed CUIC) is located in the 5′UTR of about 70% of mRNAs encoding ribosomal proteins, and this sequence is required for netrin-1 induced translation of these mRNAs in *Xenopus* retinal ganglion cell axons (Shigeoka et al., 2019). Furthermore, these newly translated ribosomal proteins can become part of the ribosomal complex in axons, and loss of nascent ribosomal proteins prevents axonal branching. TAR DNA-binding protein (TDP-43), an RBP, is also involved in this process. TDP-43 shuttles between the nucleus and the cytoplasm, and regulates the transport and translation of mRNAs in the cytoplasm (Ratti and Buratti, 2016). Specifically, TDP-43 transports mRNAs encoding ribosomal proteins to axons by binding to 5′TOP sequences within the 5′UTR (Nagano et al., 2020). This study also provided further support for Shigeoka et al. (2019) by confirming that the ribosomal protein mRNAs are locally translated in axons and integrated into ribosomes. Taken together, these studies demonstrate that the local synthesis of components of the translational machinery is emerging as a vital component of axonal translation.

#### Cytoskeletal- and Membrane-Associated Proteins

There are a number of cytoskeletal- and membrane-associated proteins, including RhoA, Par3, TC10, cofilin, and GAP-43, that are locally translated in axons. These cytoskeletal-related proteins play a key role in axon growth and pathfinding by ultimately driving forward or retracting axonal growth cones in response to guidance cues. RhoA is a small GTPase that regulates actin dynamics by inducing growth cone collapse (Kozma et al., 1997). In embryonic hippocampal neurons, RhoA transcripts localize in developing axons and growth cones (Wu et al., 2005). Sema3A results in an increase in the local translation of RhoA in DRG neurons. Functionally, Sema3A induced growth cone collapse requires the local translation of RhoA in axons.

The PAR complex, which is composed of Par3, Par6, and an atypical PKC, was originally shown to regulate the cytoskeleton during the formation of neuronal polarity (Shi et al., 2003). However, this complex localizes to growth cones and axons even after axon specification has finished, suggesting that this complex may also regulate axon elongation (Hengst et al., 2009). Interestingly, *Par3* mRNA, but not *Par6* or the *atypical PKC* mRNA, localizes in axons and is locally translated in response to netrin-1 or nerve growth factor (NGF). Knocking down only intra-axonal translation of *Par3* mRNA inhibits NGF-stimulated axon elongation. Although not directly shown in this paper, the PAR complex can act on a number of signaling effectors that ultimately affect the actin and/or microtubule cytoskeleton. This paper defines an interesting mechanism by which only one member of a complex is locally translated, which may contribute to highly specific regulation in the temporal and spatial domains.

TC10 is a small GTPase required for exocyst function, which leads to membrane expansion (Gracias et al., 2014). *TC10* mRNA is localized and locally translated within developing DRG axons, in response to NGF (Gracias et al., 2014). Furthermore, this local translation is necessary for NGF-stimulated membrane expansion and axon outgrowth. Interestingly, this study identified that a PI3K-Rheb-mTOR signaling pathway regulated the translation of both TC10 and PAR3 in response to NGF. Thus, a single signaling pathway can regulate both the cytoskeleton (via PAR3) and the membrane (via TC10). This exciting finding supports the idea that there is coordinated local translation of multiple factors that are all required for appropriate axon growth (i.e., RNA regulons).

Cofilin is an actin-depolymerizing protein that directly regulates growth cone motility (Meberg et al., 1998; Meberg and Bamburg, 2000). *Cofilin* mRNA is locally synthesized in response to the guidance cues Slit2 and Sema3A, leading to growth cone collapse in late-stage *Xenopus* retinal ganglion cells (Piper et al., 2006). This local translation of cofilin occurs preferentially in a cap-independent manner via an internal ribosome entry site (IRES) present in the 5′UTR of *cofilin* mRNA (Choi et al., 2018). Further, IRES-mediated translation of cofilin is required for axon outgrowth and growth cone steering in primary hippocampal neurons in response to the repulsive cue Sema3A. Immunoprecipitation studies show that *cofilin* mRNA interacts with *Xenopus* Vg1RBP, a homolog of ZBP1 (Piper et al., 2006), but the mechanisms underlying this interaction require further study. Together, these studies show that local translation of cofilin regulates cytoskeletal remodeling that contributes to axonal pathfinding.

GAP-43 is selectively localized to axonal growth cones during development and is involved in multiple aspects of axon development, including neuronal polarity, axon growth and axon pathfinding (Goslin et al., 1988, 1990; Strittmatter et al., 1995). *GAP-43* mRNA localizes in axons in a ZBP1-dependent manner, similar to β*-actin* mRNA (Donnelly et al., 2011). In fact, it has been shown that there is competition between β*-actin* mRNA and *GAP-43* mRNA for binding to ZBP1. hnRNP-Q1, another mRNA-binding protein, binds to the 5′UTR of *GAP-43* mRNA and represses its translation (Williams et al., 2016). Functionally, knocking down hnRNP-Q1 alters developing cortical neuron morphology, including an increase in axon length and the number of neurites. These morphological alterations can be rescued by also knocking down GAP-43, suggesting that hnRNP-Q1 tightly regulates the local translation of *GAP-43* mRNA thereby leading to appropriate neurite growth. These studies begin to show the complexity of competition between multiple mRNAs and RBPs to regulate local translation, but much more research is needed in this area. Overall, these findings indicate that the local translation of a variety of cytoskeletal- and membrane-associated mRNAs underlie axon growth and guidance.

### Broader Mechanisms Regulating Local Translation in Axons

#### Direct Interaction of Receptors With Translational Machinery

Now that local translation in developing axons has been firmly established as an important mechanism regulating axon growth and guidance, more recent studies have begun to shed light on how this process is regulated. One mechanism that regulates local translation is the direct interaction of transmembrane receptors, such as Deleted in colorectal cancer (DCC), with ribosomal proteins and other translation machinery (Tcherkezian et al., 2010; Koppers et al., 2019). In spinal commissural axons, DCC colocalizes with ribosomes within growth cones and this complex is specifically enriched in the tips of filopodia (Tcherkezian et al., 2010). DCC also co-precipitates with many translation initiation factors and ribosomal subunits. Stimulation with netrin-1, which binds to DCC, leads to the release of ribosomal subunits from DCC, resulting in the progressive formation of polysomes and increased local translation (Tcherkezian et al., 2010). This study was the first to demonstrate a mechanism through which extracellular guidance cues could directly activate local translation at transmembrane receptor sites. It has yet to be shown that netrin-1 induced local translation of β*-actin* is mediated directly through DCC-associated translational machinery, but this is an interesting area for further study. A more recent study has built on these findings to demonstrate that receptor-ribosome coupling is a widespread process, that is, there are a number of transmembrane receptors, including DCC, Neuropilin-1 and Robo2, that are directly associated with translational machinery (Koppers et al., 2019). Furthermore, this is a specific process in that certain guidance cue-receptor signaling activates the translation of one set of mRNAs, whereas a different guidance cue-receptor complex activates the translation of a different set of mRNAs. It will be important in the future to better understand how mRNAs are translated under various stimulated conditions, either through direct interactions of receptors with translational machinery or via receptors activating intracellular signaling cascades that then lead to translation.

#### Signaling Pathways

Specific signaling pathways are involved in the local translation of multiple mRNAs in axons. For example, the mTOR pathway plays a central role in this process. NGF-induced activation of the PI3K-Rheb-mTOR signaling pathway regulates local translation of *Par3* and *TC10* mRNAs during axon outgrowth of DRG neurons (Gracias et al., 2014). mTOR and MAPK are also involved in Slit-induced protein synthesis-dependent growth cone collapse of retinal ganglion cells (Piper et al., 2006). In addition, the PI3K-Akt-mTOR signaling pathway is needed for netrin-1 induced local protein synthesis in growth cones (Piper et al., 2015). The mTOR signaling cascade can regulate translation through multiple pathways, including the phosphorylation of eIF4E-binding proteins (4E-BPs) and p70 ribosomal S6 protein kinase 1 (Laplante and Sabatini, 2012; Switon et al., 2017). mTOR itself is also locally translated in injured axons (Terenzio et al., 2018), suggesting an interesting area of future study for developing axons.

#### Regulation of Translation by miRNAs and mRNA Modifications

Non-coding RNAs, such as microRNAs (miRNAs), are present in axons and regulate translation. Numerous miRNAs have been identified in the axons of multiple cell types, including *Xenopus* retinal ganglion cells and mouse cortical neurons (Sasaki et al., 2014; Bellon et al., 2017). Due to space limitations, we only highlight a few miRNA studies herein; we direct the reader to a recent review for more information on this subject (Corradi and Baudet, 2020). In *Xenopus* retinal ganglion cell axons, miR-182 was identified as the most enriched, and further study demonstrated that it regulates local translation during axon pathfinding (Bellon et al., 2017). Under basal conditions, miR-182 binds to *cofilin* mRNA in growth cones and represses its translation. However, Slit2 binding to Robo2/3 results in the release of miR-182 repression, translation of cofilin and growth cone repulsion. Functionally, loss of miR-182 impairs Slit2 regulated axon guidance and targeting both *in vitro* and *in vivo*. This paper (Bellon et al., 2017) showed that a guidance cue, Slit, could relieve miRNA inhibition and stimulate translation. However, the reverse has also been shown. Guidance cues can result in the activation of miRNAs, which then bind to mRNAs and repress translation. Another study, also in retinal ganglion cell growth cones, demonstrated that Sema3A stimulation results in the processing of specific pre-miRNAs to miRNAs and subsequent translational repression of *TUBB3* mRNA (Corradi et al., 2020). Interfering with the mature miRNAs results in disrupted *in vivo* axon guidance. Taken together, these studies show that miRNAs regulate local translation through a variety of mechanisms.

Much is still unknown about the role of non-coding mRNAs during axon development. For instance, a recent study found that ALAE, a long intergenic non-coding RNA (lincRNA), is enriched in DRG axons during axon elongation and regulates local translation during this stage of development (Wei et al., 2021). More recent findings also suggest that there can be direct modifications of mRNAs to regulate translation. *N*^6^-methyladenosine (m^6^A) is a reversible modification of mRNA that can affect its translational status. Interestingly, an m^6^A eraser, FTO, is in axons and can alter the m^6^A levels of *GAP-43* mRNA (Yu et al., 2018). When axonal FTO levels are reduced in axons, this leads to an increase in the m^6^A levels of *GAP-43* mRNA, decreased translation of *GAP-43* mRNA, and decreased axon outgrowth. Further, an m^6^A reader, YTHDF1, regulates the local translation of the axon guidance receptor *Robo3.1* mRNA in spinal commissural neurons (Zhuang et al., 2019). Loss of YTHDF1 results in a significant reduction in the local translation of Robo3.1 and axon guidance deficits. Thus, direct modifications of RNAs, including m^6^A, in axons is an emerging area of study, and may be an important modulator of local translation.

#### Organelles and Mitochondria

Late endosomes mediate both the localization of the translation complex and local translation in axons (Cioni et al., 2019). Ribosomes, RBPs, and mRNAs, including β*-actin* mRNA, are localized on late endosomes. This local translation complex “hitchhikes” on the endosome for transport in axons. Furthermore, the late endosomes are sites for local translation, and this process is important for mitochondrial function. Local translation on late endosomes often occurs when the endosomes have paused on mitochondria. Thus, this study identifies a novel mechanism and provides new information about how and where local translation can occur in axons to support ongoing cellular function. In particular, the role of mitochondria in local translation is a rapidly emerging area of investigation, and future studies will surely provide new insights into this and other mechanisms that regulate translation within developing axons.

## Local Translation During Axon Branching

During development, axons generate multiple filopodia that mature into branches and connect with their target neurons, forming synapses. This process is necessary for the formation of a complex neuronal circuit. Guidance cues, including netrin-1, NGF, and BDNF, that are involved in growth cone steering also promote axonal branching (Dent et al., 2004; Tang and Kalil, 2005; Marler et al., 2008; Spillane et al., 2012). One mechanism that regulates this branching is local translation mediated by RBPs, such as FMRP and Vg1RBP (ZBP1) (Pan et al., 2004; Tucker et al., 2006; Kalous et al., 2014; Wong et al., 2017). Here, we review key studies demonstrating that local translation regulates axon branching in the developing nervous system.

β-actin plays an important role in the formation and stabilization of axonal arbors. The *Xenopus* homolog of ZBP1 (Vg1RBP), which is an RBP that binds to β*-actin* mRNA, was first shown to be enriched at the base of filopodia within axonal shafts (Kalous et al., 2014). Using high-resolution live-cell imaging and translational reporter analysis, further studies in *Xenopus* retinal ganglion cells revealed that β*-actin* mRNA and RNP granules co-dock at branch points with mitochondria (Wong et al., 2017). These sites stimulate branch formation through the synthesis of nascent β-actin in response to external stimuli (Kalous et al., 2014; Wong et al., 2017). Moreover, knockdown of Vg1RBP results in fewer axonal filopodia and branches, and inhibiting the translation of β*-actin* mRNA reduces the arbor complexity of *Xenopus* retinal ganglion cells (Kalous et al., 2014; Wong et al., 2017). Together, these data suggest that local translation of β-actin is critical to the process of axonal branching (Figure 2).

Other components involved in the regulation of actin cytoskeleton are also locally translated at branch points and initiate axon branching. Using chicken embryonic sensory neurons, Spillane et al. (2012) showed that NGF induces the formation of axonal actin patches, filopodia, and branching in a local protein synthesis-dependent manner (Spillane et al., 2012). WAVE1 activates the Arp2/3 complex, an actin-nucleating complex that is involved in axonal branching, and cortactin stabilizes these Arp2/3-mediated actin filaments. This study demonstrates that NGF results in local protein synthesis of WAVE1 and cortactin, which then contributes to axonal branching. Interestingly, NGF induces axonal branching at sites that are enriched with mitochondria (Spillane et al., 2013). ZBP1, ribosomes, and mitochondria are all enriched at the base of axonal filopodia. Stalling of mitochondria at the base of axonal filopodia, in response to NGF treatment, results in hotspots of local translation and the formation of a stable axon branch. Thus, NGF stimulation results in the enrichment of mitochondria and other translational machinery at the axonal branch points, concentrated local translation, and the subsequent maturation of filopodia into axonal branches.

## Local Translation During Synaptogenesis

Synaptogenesis is a key step in the formation of a functional nervous system and regulated local translation of mRNAs at both pre- and post-synaptic sites contributes to this process. Synaptosomal-associated protein 25 (SNAP25) is a t-SNARE protein necessary for synaptic vesicle exocytosis. Interestingly, when the axons of developing hippocampal neurons come into contact with poly-D-lysine coated beads, initiating presynapse formation, the localization and local translation of *SNAP25* mRNA is stimulated (Batista et al., 2017). This process is thought to contribute to the clustering of presynaptic proteins, thereby regulating the assembly of presynaptic terminals. Functionally, inhibiting the intra-axonal synthesis of SNAP25 reduces presynaptic vesicle release, and thus likely interferes with proper synaptic function.

Similarly, poly-D-lysine coated beads stimulate the local translation of β-catenin, a protein that also contributes to presynaptic vesicle release (Taylor et al., 2013; Batista et al., 2017). Axon-specific knockdown of β*-catenin* mRNA leads to impaired presynaptic vesicle release (Taylor et al., 2013). However, local translation of β-catenin is required only during the initial steps of presynaptic assembly, unlike SNAP25, which is continually required until at least 12 h after the initiation of presynaptic assembly (Batista et al., 2017). These findings suggest that β-catenin is locally translated in small amounts and the locally translated population is needed only for the initial clustering stage; additional β-catenin needed after that point is provided by anterograde protein transport from the cell body. This is in contrast with SNAP25, which is locally translated in much higher amounts and is likely required for both initial clustering of presynaptic proteins and continued maintenance of presynaptic terminals and release of synaptic vesicles in mature synapses.

Munc18-1 is a presynaptic protein involved in neurotransmitter release, and leucine-rich repeat transmembrane neuronal 2 (LRRTM2) is a presynaptic organizer. Interestingly, Munc18-1 is locally translated in mouse cortical neuron presynaptic terminals exposed to LRRTM2-conjugated beads (Parvin et al., 2019). Further, Munc18-1 and the mRNA binding protein, FMRP, colocalize in the induced presynaptic terminals. Knockout of FMRP increases the accumulation of Munc18-1 at the presynaptic boutons, suggesting that FMRP regulates the local translation of the Munc18-1 at nascent presynaptic sites (Parvin et al., 2019). Munc18-1 regulates calcium-dependent neurotransmitter release. Thus, an increase in Munc18-1 expression due to loss of FMRP can lead to impaired presynaptic functions and excessive neurotransmission. This could potentially contribute to cognitive impairment or other phenotypes of Fragile X syndrome, a disorder caused by trinucleotide repeat expansions in the *FMR1* gene that leads to loss of FMRP.

More recent studies have also focused on how translation machinery is regulated over the time course of neuronal development. Specifically, the levels of axonal ribosomes appear to be tightly regulated in the process that leads to axonal maturation (Costa et al., 2019). Interestingly, this study demonstrates that the ribosomal levels in axons of developing rat hippocampal neurons significantly decrease following synapse formation, thereby suggesting that synaptogenesis is a hallmark for the reduction. It is important to note that despite this reduction, local protein synthesis in mature axons is also vital for their function, although this topic is outside the scope of this review (for reviews on this topic, see Sahoo et al., 2018; Kim and Jung, 2020). Taken together, these data indicate that local protein synthesis is emerging as a critical mechanism regulating synaptogenesis, although much is still unknown about the mRNAs that are locally translated and the mechanisms regulating this process.

## Local Translation in Neurological Diseases

Neurodevelopmental disorders can result from impairments in neuronal differentiation, migration, axon growth and guidance, and/or synaptogenesis. As highlighted in this review, mRNA localization and local translation of proteins are essential for each of these developmental stages. Studies in this area are consistently emerging and will help identify potential therapeutic targets for neurodevelopmental disorders. In this section, we highlight important studies that provide insight into the contribution of dysfunctional local translation to neurological disorders (summarized in Table 1).

**TABLE 1:** Altered local translation in neurological disorders.

Neurological disorder	Functional consequence	Possible translational mechanism in axons	Developmental process potentially affected	References
Fragile X syndrome	Reduced Sema3A-induced growth cone collapse in *Fmr1* knockout hippocampal neurons	Sema3A-induced translation of *Map1B* mRNA; *Map1B* mRNA is bound by FMRP	Axon growth and guidance	Antar et al., 2006; Li et al., 2009; Takabatake et al., 2020
Fragile X syndrome	Unknown	FMRP-mediated translation of *Munc18-1* mRNA	Synaptogenesis	Parvin et al., 2019
Fragile X syndrome	Impaired NGF-induced axonal elongation in FMRP knockdown or miR-181d overexpressing neurons	FMRP-miR-181d interaction regulates translation of *Map1B* and calmodulin mRNAs	Microtubule stabilization during axon elongation	Wang et al., 2015
Down syndrome, Fragile X syndrome and Intellectual disability	DSCAM overexpression results in decreased axon length in mouse hippocampal neurons and impaired synaptic targeting in *Drosophila* FMRP knockout animals	*Dscam* mRNA colocalizes with FMRP and CPEB1 (hippocampal neurons); FMRP binds to *Dscam* mRNA (Drosophila); DSCAM is locally translated in growth cones (hippocampal neurons)	Axon growth and synaptogenesis	Cvetkovska et al., 2013; Jain and Welshhans, 2016
ASD and Intellectual disability	Loss of APC results in reduced social interest and repetitive behavior in mice	APC binds to β*2B-tubulin* and β*-catenin* mRNAs	Neuron migration and Axon outgrowth	Barth et al., 2008; Fortress et al., 2013; Mohn et al., 2014; Preitner et al., 2014
ASD and Down syndrome	Unknown	Mena is necessary for the local translation of *Dyrk1a*	Axon growth and guidance	Vidaki et al., 2017
Charcot-Marie-Tooth Disease	Expression of CMT2b Rab7a mutants results in deficits in axon guidance and viability	Rab7a endosomes carry mRNAs and are sites of local protein synthesis	Axon growth and guidance	Cioni et al., 2019
Spinal Muscular Atrophy	Impaired axon outgrowth in SMA mouse model	Loss of SMN results in mislocalization of β*-actin* and *GAP-43* mRNAs	Axon outgrowth	Rossoll et al., 2003; Fallini et al., 2016

### Fragile X Syndrome (FXS), Autism Spectrum Disorder (ASD), and Down Syndrome

FXS, an X-linked disorder, is the most prevalent inherited cause of intellectual disability and the most common monogenic cause of ASD. It results from the loss of FMRP, a protein encoded by the *FMR1* gene (reviewed in Bagni et al., 2012). The 5′UTR of *FMR1* contains a variable number of trinucleotide CGG repeats (Oberlé et al., 1991; Verkerk et al., 1991). The modal number of CGG repeats in humans is 30. However, expansion of these repeats to more than 200 copies leads to methylation, transcriptional silencing of *FMR1* (Sutcliffe et al., 1992), and the loss of FMRP. FMRP is an mRNA binding protein that regulates translation via multiple mechanisms, including ribosome stalling (Darnell et al., 2011; Chen et al., 2014). FMRP is localized throughout neurons, including in the axons and growth cones of developing neurons (Antar et al., 2006). Although the function of FMRP in dendrites is well studied, comparatively few studies have examined the contribution of axonal FMRP to FXS. However, these studies have demonstrated that it regulates the local protein synthesis of axonal mRNAs, thereby affecting the composition of the axonal proteome. Functionally, loss of FMRP alters multiple neurodevelopmental processes, including neurogenesis and axon targeting (Li et al., 2009; Luo et al., 2010; Li and Zhao, 2014; Halevy et al., 2015; Richter et al., 2015; Lai et al., 2020).

Multiple studies using high-throughput approaches have identified thousands of FMRP target mRNAs. FMRP generally functions as a translational repressor for its mRNA targets and, thus, loss of FMRP leads to excessive translation. Many of these target mRNAs, including neuroligin 3, *SHANK3*, and neurexin 1, are associated with ASD (Darnell et al., 2011; and reviewed in Bagni and Zukin, 2019). Neuroligins and neurexins are synaptic cell adhesion molecules (Nguyen and Südhof, 1997; Patel et al., 2015) and SHANK3 is a protein localized to the postsynaptic density (Arons et al., 2016); together these proteins contribute to synaptogenesis (Arons et al., 2012). As discussed in the section on neurogenesis earlier in this review, FMRP also binds numerous mRNAs in radial glial cells, however, their role in radial glial cell endfeet and how dysregulation of this process may contribute to FXS and ASD is unknown. This is of interest because loss of FMRP accelerates neurogenesis and neurodifferentiation in specific populations of neurons, and appropriate brain development depends on these developmental steps occurring with precise timing (Castren, 2016; Bardoni et al., 2017). Altered neurogenesis and neurodifferentiation timelines that lead to inappropriate brain maturation could contribute to clinical phenotypes of FXS, which include cognitive impairments in executive function and short term memory (Huddleston et al., 2014).

In the context of axon guidance and synaptogenesis, FMRP regulates the translation of specific mRNAs, including MAP1B, in response to Sema3A stimulation of mouse hippocampal axons (Antar et al., 2006; Li et al., 2009). Furthermore, Sema3A leads to the degradation of FMRP in growth cones of developing cortical neurons, a process mediated by ubiquitination (Takabatake et al., 2020). Thus, it may be that Sema3A results in the degradation of FMRP, thereby leading to the local translation of MAP1B. Functionally, Sema3A causes growth cone collapse, but this is reduced in *Fmr1* knockout hippocampal neurons (Li et al., 2009). *Fmr1* knockout neurons also show an increase in filopodial density and reduced motility, as compared to wild type neurons (Antar et al., 2006). Finally, presynaptic function is also impaired in *Fmr1* knockout neurons (Deng et al., 2011). As mentioned in the synaptogenesis section, FMRP regulates the local translation of Munc18-1, a synaptic vesicle fusion protein that is necessary for presynapse formation (Parvin et al., 2019). This finding is relevant to FXS pathology because excessive Munc18-1 translation in presynapses is thought to lead to excessive glutamate release during synaptogenesis (Parvin et al., 2019). This excessive neurotransmitter release would cause impaired formation of synaptic connections and the altered synaptic phenotypes of FXS, including changes in synaptic physiology and plasticity (Bagni and Zukin, 2019).

A recent study nicely complemented these findings to reveal that FMRP binds to a non-coding RNA, miR-181d, in the axons of embryonic DRG neurons (Wang et al., 2015). Interestingly, both FMRP and miR-181d bind to *Map1b* and calmodulin (*Calm1*) mRNAs. NGF stimulation results in the release of *Map1b* and *Calm1* mRNAs from FMRP granules, their local translation, and an increase in axon elongation. FMRP deficiency disrupts this process. Together, these findings indicate that miR-181d negatively regulates the translation of MAP1B and Calm1, thereby playing a role in axon elongation via microtubule stabilization and calcium signaling. Thus, FMRP regulates cytoskeletal changes in axons and growth cones during development by interacting with non-coding mRNAs, and disruption of this process may contribute to FXS. Specifically, these types of changes in axon motility could result in altered axonal connectivity, which occurs in multiple brain regions of FXS patients (Bagni and Zukin, 2019).

Another potential target of FMRP is *Dscam* mRNA, which is locally translated in growth cones in response to the guidance cue netrin-1 (Jain and Welshhans, 2016). In embryonic hippocampal growth cones, *Dscam* mRNA was found to be colocalized with FMRP and another mRNA-binding protein, CPEB1. Furthermore, overexpression of DSCAM, which mimics the Down syndrome condition, resulted in decreased axon length (Jain and Welshhans, 2016). Overexpression of *Dscam* also occurs in *Drosophila* FMRP knockout animals and leads to improper synaptic targeting (Cvetkovska et al., 2013). These studies provide a link between two neurodevelopmental disorders, FXS and Down syndrome, both of which are associated with intellectual disability (Jain and Welshhans, 2016).

Disruptions in cytoskeletal dynamics, which directly regulate growth cone motility, have also been associated with neurodevelopmental disorders. The assembly and disassembly of actin polymers is necessary for appropriate growth cone pathfinding (Rodriguez et al., 2003; Dent et al., 2011; Vitriol and Zheng, 2012; Omotade et al., 2017). Thus, changes in local translation of actin or actin-regulating proteins could contribute to neurological disorders, such as periventricular heterotopia, a malformation of cortical development that is due to disruption of the actin cytoskeleton (Lian and Sheen, 2015; Tang and Jin, 2018). Although local translation has not been implicated in this disorder, much is still unknown about this and other related disorders. In terms of microtubules, Adenomatous Polyposis Coli (APC) promotes microtubule assembly at the plus-end, thereby regulating cell migration and polarization (Barth et al., 2008; Preitner et al., 2014). APC binds to mRNAs such as β*2B-tubulin* and β*-catenin* and regulates their localization within the growth cone periphery (Preitner et al., 2014). Altered β-catenin levels lead to impaired memory consolidation (Fortress et al., 2013; Mohn et al., 2014). Loss of APC leads to autism-like phenotypes in mice, including reduced social interactions and an increase in repetitive behavior (Mohn et al., 2014). Thus, these and other studies suggest a link between altered APC and ASD and/or intellectual disability (Hodgson et al., 1993; Raedle et al., 2001; Finch et al., 2005; Heald et al., 2007; Kumar et al., 2019). Further, Dual Specificity Tyrosine Phosphorylation Regulated Kinase 1A (*DYRK1A*), a gene implicated in both ASD and Down syndrome, is locally translated within axons of developing mouse cortical neurons (Vidaki et al., 2017). This study showed that *Dyrk1a* mRNA forms a complex with *Mena*, an actin regulator, and loss of *Mena* disrupts the local translation of *Dyrk1a* mRNA. In summary, local protein synthesis of cytoskeletal and cytoskeletal-related proteins is necessary for neuronal development. Impairment of neurogenesis and axonal pathfinding due to cytoskeletal changes could be associated with connectivity deficits, leading to intellectual disability or other changes in neuronal networks underlying neurodevelopmental disorders.

Besides mRNA transcripts, mutations in the other components of the translation machinery, such as the eukaryotic initiation factors eIF4E (cap-binding protein), 4E-BP2 (4E-binding protein), and eIF4G (interacting partner of eIF4E), can disrupt local protein synthesis (Chen et al., 2019). Knockdown of 4E-BP2 or overexpression of eIF4E leads to an increase in neuroligins (Gkogkas et al., 2013), proteins that are locally translated (Chmielewska et al., 2019) and required for synaptogenesis as discussed above. Furthermore, overexpression of eIF4E in mice results in increased cap-dependent translation and leads to perturbed synaptic transmission (Santini et al., 2013). These mice also show behavioral similarities to autism, including social interaction deficits. More research is needed to specifically understand how changes in local translation machinery contribute to changes during development. However, it has been established that regulated translation by eIFs is critical to maintaining physiological functions during development and thus, may be potential targets in developing therapeutics for neurodevelopmental disorders, including ASD.

### Charcot-Marie-Tooth Disease (CMT)

Charcot-Marie-Tooth disease is a neurodegenerative disorder that is caused by defects in Schwann cells and/or peripheral nerves. Because more than 100 different gene mutations have been linked to CMT, this disorder has multiple inheritance patterns, including autosomal dominant, autosomal recessive, and X-linked dominant (Pipis et al., 2019). Here we focus on specific mutations in *Rab7a*, which are associated with the CMT2b subtype (Verhoeven et al., 2003; Meggouh et al., 2006; Spinosa et al., 2008; Wang et al., 2014). Endosomes transport proteins and lipids to their targets within neurons. Cioni et al. (2019) observed that in *Xenopus* retinal ganglion cells, Rab7a endosomes carry RNPs along axons (Cioni et al., 2019). The endosomal proteins, Rab5a and Rab7a, colocalize with ribosomal proteins, Vg1RBP (ZBP1), and β*-actin* mRNA, within axons. Further, these endosomes are enriched at mitochondria, suggesting that endosomes could be a site for local translation of mRNAs that are essential for mitochondrial function. Interestingly, when a CMT type 2B Rab7a mutant was expressed in retinal ganglion cells, multiple impairments resulted, including deficits in axonal growth and guidance, loss of local protein synthesis, and altered mitochondrial function. Thus, impaired local translation due to mutations in *Rab7a* is a possible mechanism underlying some of the deficits seen in CMT2b.

### Spinal Muscular Atrophy (SMA)

Spinal muscular atrophy is a motor neuron disease that is caused by mutations in the survival of motor neuron 1 gene (*SMN1)*, resulting in reduced levels of SMN protein. SMN is an mRNA binding protein and key member of the spliceosome. Small nuclear ribonucleoproteins (snRNPs) complex with other proteins to form the spliceosome and carry out pre-mRNA splicing (Singh et al., 2017). SMN is localized within axons and growth cones of motor neurons both *in vitro* and *in vivo* (Jablonka et al., 2001; Fan and Simard, 2002). In the SMA mouse model, expression of heterogeneous nuclear ribonucleoprotein (hnRNP), a binding partner of SMN, and β-actin are significantly reduced in motor axons (Rossoll et al., 2003). Furthermore, growth cone area of motor neurons is reduced and compartmentalized localization of mRNA transcripts and local translation in growth cones of cortical neurons is also impaired (Fallini et al., 2016). Specifically, there is a severe reduction in β*-actin* and *Gap-43* mRNAs and proteins within growth cones (Rossoll et al., 2003; Fallini et al., 2016). However, the deficits in axon outgrowth in the SMA mouse model can be rescued by overexpressing mRNA binding proteins that result in increased local translation of GAP-43. Together, these findings suggest that the local synthesis of actin regulators controls the formation of the neuronal framework and its dysregulation is implicated in SMA. The main pathophysiology of SMA is degeneration of lower motor neurons, thus it is not clear how deficits in developmental axon growth contribute to SMA. However, GAP-43 also regulates mature axon maintenance and presynaptic stability (Grasselli et al., 2011; Allegra Mascaro et al., 2013); thus, a continued disruption in local translation beyond early development could contribute to the degeneration of lower motor neurons in SMA.

## Summary and Discussion

Our current knowledge about the locally translated proteome and its reorganization during development establishes that local translation is a complex phenomenon that is critical to the development of the nervous system. Here, we have highlighted the key players that facilitate local protein synthesis throughout development. However, local translation within radial glial cells is a relatively new niche and only a few subsets of mRNAs have been identified to date. More work in this field is required to identify other transcriptomes that regulate neuronal differentiation and migration. With the recent development and increasing use of high throughput screening techniques, including HITS-CLIP and RNA-seq, many new mRNA datasets are being progressively identified in the subcellular domains of developing neurons, but their functions are not well understood or studied. In addition, most of these findings use *in vitro* approaches, and therefore there is a gap in our knowledge about whether these findings will be confirmed when tested *in vivo*.

Recent studies have begun to advance our knowledge about the molecular mechanisms regulating local translation in developing axons, and how this process is coordinated between functionally related mRNAs. Emerging techniques will advance the local translation field and provide more information about the multiple mechanisms that regulate local translation, both from a systematic and functional perspective. For example, optogenetic control of mRNA localization and translation can be achieved through mRNA-LARIAT (mRNA-light-activated reversible inactivation by assembled trap) (Kim et al., 2020). This is a powerful technique that can control mRNA localization and translation with high spatial and temporal resolution. When combined with the RCas9 system, it can also be used to manipulate endogenous mRNAs. Single molecule imaging has also started to provide novel and exciting information about mRNA dynamics and local protein synthesis, but needs to be applied more extensively (Ströhl et al., 2017; Turner-Bridger et al., 2018; Boersma et al., 2019; Mateju et al., 2020; Donlin-Asp et al., 2021). Taken together, the studies reviewed herein demonstrate a key role for mRNA localization and local translation during multiple stages of neural development. Furthermore, they highlight the need to better understand how dysregulation of this process contributes to various neurodevelopmental disorders.

## Author Contributions

MA and KW conceived the idea for this review, reviewed the literature, and edited and revised the manuscript and figures. MA wrote the first draft of the manuscript, including the figures. Both authors contributed to the article and approved the submitted version.

## Conflict of Interest

The authors declare that the research was conducted in the absence of any commercial or financial relationships that could be construed as a potential conflict of interest.

## Publisher’s Note

All claims expressed in this article are solely those of the authors and do not necessarily represent those of their affiliated organizations, or those of the publisher, the editors and the reviewers. Any product that may be evaluated in this article, or claim that may be made by its manufacturer, is not guaranteed or endorsed by the publisher.
